# Evaluation of Algal Biofilms on Indium Tin Oxide (ITO) for Use in Biophotovoltaic Platforms Based on Photosynthetic Performance

**DOI:** 10.1371/journal.pone.0097643

**Published:** 2014-05-29

**Authors:** Fong-Lee Ng, Siew-Moi Phang, Vengadesh Periasamy, Kamran Yunus, Adrian C. Fisher

**Affiliations:** 1 Institute of Ocean and Earth Sciences, University of Malaya, Kuala Lumpur, Malaysia; 2 Institute of Biological Sciences, Faculty of Science, University of Malaya, Kuala Lumpur, Malaysia; 3 Low Dimensional Materials Research Centre, Department of Physics, Faculty of Science, University of Malaya, Kuala Lumpur, Malaysia; 4 Centre of Research for Electrochemical, Science and Technology (CREST), Department of Chemical Engineering and Biotechnology, University of Cambridge, Cambridge, United Kingdom; US Naval Reseach Laboratory, United States of America

## Abstract

In photosynthesis, a very small amount of the solar energy absorbed is transformed into chemical energy, while the rest is wasted as heat and fluorescence. This excess energy can be harvested through biophotovoltaic platforms to generate electrical energy. In this study, algal biofilms formed on ITO anodes were investigated for use in the algal biophotovoltaic platforms. Sixteen algal strains, comprising local isolates and two diatoms obtained from the Culture Collection of Marine Phytoplankton (CCMP), USA, were screened and eight were selected based on the growth rate, biochemical composition and photosynthesis performance using suspension cultures. Differences in biofilm formation between the eight algal strains as well as their rapid light curve (RLC) generated using a pulse amplitude modulation (PAM) fluorometer, were examined. The RLC provides detailed information on the saturation characteristics of electron transport and overall photosynthetic performance of the algae. Four algal strains, belonging to the Cyanophyta (Cyanobacteria) *Synechococcus elongatus* (UMACC 105), *Spirulina platensis*. (UMACC 159) and the Chlorophyta *Chlorella vulgaris* (UMACC 051), and *Chlorella* sp. (UMACC 313) were finally selected for investigation using biophotovoltaic platforms. Based on power output per Chl-a content, the algae can be ranked as follows: *Synechococcus elongatus* (UMACC 105) (6.38×10^−5^ Wm^−2^/µgChl-a)>*Chlorella vulgaris* UMACC 051 (2.24×10^−5^ Wm^−2^/µgChl-a)>*Chlorella* sp.(UMACC 313) (1.43×10^−5^ Wm^−2^/µgChl-a)>*Spirulina platensis* (UMACC 159) (4.90×10^−6^ Wm^−2^/µgChl-a). Our study showed that local algal strains have potential for use in biophotovoltaic platforms due to their high photosynthetic performance, ability to produce biofilm and generation of electrical power.

## Introduction

Algae are amongst the most efficient photosynthetic organisms with fast growth rates, diverse products and tolerance to extreme environments. Diatoms, green algae and cyanobacteria (also referred to as the blue-green algae, Cyanophyta) are the major primary producers in the aquatic ecosystem, contributing to carbon dioxide removal, photo-oxygenation and also serving as sources of valuable biochemicals [1]. The biomass productivity of microalgae was estimated to be 50 times higher than switchgrass, which is the fastest growing terrestial plant [2]. With the increased interest in alternative energy sources, algae are being investigated as feedstock for biodiesel, bioethanol, biohydrogen and bioelectricity production [3], [4], [5]. Microalgae have oil content exceeding 80% DW, grow fast and can be mass cultured using open ponds or enclosed photobioreactors [6]. The filamentous cyanobacterium *Anabaena* was reportedly the first to be used for hydrogen generation [7], [8]. In 1997, electricity was generated using 2-hydroxy-1,4-naphthoquinone as an electron shuttle between *Synechococcus* sp. (UTEX 2380) and a carbon-cloth anode [9]. The current intensity increased with increasing cell concentration, reaching 320 µAcm^−2^ [9]. A bioreactor (microbial fuel cell) with an air cathode and a graphite-felt anode coated by a biofilm of bacteria and algae, generated electricity when irradiated. On day 10, the voltage output and current density produced by the reactor were 0.32 V and 8.6 µAcm^−2^ respectively [10].

Biological components have since then been introduced into fuel cells (FCs), giving rise to microbial fuel cells (MFCs). MFCs were designed to operate as a new generation of solar cells called biological photovoltaic devices (Biophotovoltaic, BPV) [11]. Both the MFCs and BPV share similar functions; where MFCs generate electricity from the metabolic process of living microbes, whereas BPV produce electricity from light energy via the light harvesting apparatus of photosynthetic organisms [12]. Previous BPV studies have utilized various exogenous soluble mediator compounds to facilitate electron transfer such as 5 mM ferricyanide as mediator between the biological materials and the anode [11]. In their paper, a new BPV device was fabricated with several advantages over the previous experiments such as multiple microchannels to facilitate multiple simultaneous experiments, removal of the need for external energy supply and a new design to cater for investigation with various types of biological materials instead of solely a single type. Results indicated a direct relationship between effects of cell density, electron mediator concentration and light intensity with the efficiency of the BPV device. The biological materials used in this study were intact *Synechocystis* cells and thylakoid membranes isolated from the cells, which generated total power output of 4.71 and 9.28 nWµmol Chl^−1^ respectively. In another study, McCormick et al. [13] replaced the use of exogenous mediators with the development of biofilms. Several algae were grown directly on an Indium tin oxide- Polyethylene terephthalate (ITO-PET) anode on a sandwich type or an open air design. This work demonstrates the ability to produce simple, portable BPV devices without the need of an artificial electron mediator. The *Synechococcus* biofilm in this work produced a peak power output at 1.03×10^−2^ Wm^−2^ under 10 Wm^−2^ of white light [13]. Biofilm development offers several advantages by increased power output due to direct contact between cell and electrode and reduced internal potential losses. In 1684, Antonie van Leeuwenhoek [14] reported the existence of animalcules on teeth, representing the first scientific report on biofilms. Microorganisms are able to attach to surfaces and form a hydrated polymeric matrix termed “extra-cellular polymeric substances (EPS)” that hold the biofilm together [15]. In an EPS matrix, there are interstitial water channels, which separate bacterial cells from each other, thereby allowing the transportation of nutrients, oxygen and genes [16]. Biofilms composed of microorganisms attached to surfaces, form a hydrated polymeric matrix consisting of polysaccharides, protein and nucleic acids [15]. There is a growing interest in the study of artificial phototrophic biofilms. In biofuel production, the cultivation of algae as biofilms reduces costs due to reduction of water volume and power required for pumping. This increases biomass and also avoids costly harvesting and dewatering technologies [17]. Traditional methods of monitoring changes in biomass of microphytobenthic biofilms are destructive, for example, pigment sampling by using syringe coring method. This method involves pulling the syringe plunger up while pushing the syringe to the sediment to create counter pressure [18]. Fluorescence techniques have been employed to measure photosynthetic performance of biofilms in sediments without interfering with the sediment surface [19]. Radiant energy absorbed by chlorophyll can undergo one of three fates: (i) used for photosynthesis (ii) dissipated as heat or (iii) re-emitted as chlorophyll fluorescence [20]. Hence, by measuring the yield of chlorophyll fluorescence, information about the efficiency of photochemistry and heat dissipation can be generated using a pulse amplitude modulation (PAM) fluorometer (Diving PAM, Walz, Germany) [21]. Photosynthesis parameters such as electron transport rate (ETR) can be calculated to measure the efficiency of photochemistry of Photosystem II (PSII) [22].

In a recent paper, Luimstra et al. [23] produced a cost effective microbial fuel cell design that can be used on algae and cyanobacteria. Several strains of benthic cyanobacteria were screened and displayed electrogenic qualities suitable for microbial fuel cell purpose. The paper also describes a particularly good green alga, *P. pseudovolvox* that demonstrated greater electrogenic activity compared to the others. However, they also highlighted that the mechanisms used in the microorganisms to donate electrons have to be determined at this moment of time. Ng et al. [24] reported that *Chlorella* sp. (UMACC 313) and *Spirulina platensis* (UMACC 159) are able to form biofilms on ITO anode. *Chlorella* and *Spirulina* formed biofilms with coverage and maximum relative electron transport rate (rETRmax) of 99.46% and 140.796 µmol electrons m^−2^ s^−1^ and 80.70% and 153.507 µmol electrons m^−2^ s^−1^ respectively. Results indicate the potential for generating electrical energy from these microalgae using biophotovoltaic platforms.

The objectives of the present study are to (i) establish libraries of 16 strains of algae for selection of most suitable strains for application in biophotovoltaic platforms; (ii) investigate biofilm formation of selected algae on Indium tin oxide (ITO) and glass and determine the photosynthetic efficiency of the biofilms; (iii) Produce and test biofilms on Indium tin oxide (ITO) in a biophotovoltaic (BPV) device for electric power generation. Glass and ITO were chosen as substrates for biofilm formation to compare the adhesion of algae cells to these surfaces. Glass is hydrophilic with high surface energies and ITO is hydrophobic with low surface energy respectively [25]. ITO was an early favorite for hole injection cathode with good transparency and conductivity [26]. ITO was also successfully used to cultivate healthy biofilms for MFCs application [13].

## Materials and Methods

### Ethics statement

Not relevant. Only microalgae were used in this study.

### Algae cultures

Fourteen local tropical algal strains from the University of Malaya Algae Culture Collection (UMACC) [27] and two strains from the Culture Collection of Marine Phytoplankton (CCMP), USA were screened for growth rate, biochemical composition and photosynthetic performance to be used for compiling the algal libraries of potential candidates for BPV platforms. The Cyanobacteria are treated as blue-green algae and will be referred to as Cyanophyta in this paper. All cultures were grown in Bold's Basal Medium for Chlorophyta, the green algae [28], Prov Medium for marine algae [27], Kosaric Medium for Cyanophyta, the blue-green algae [27] and f/2 Medium [29] for marine diatoms (Table 1). An inoculum size of 20%, standardized at an optical density at of 0.2 at 620 nm (OD_620 nm_) from exponential phase cultures was used. The cultures were grown in 1 L conical flasks in an incubator shaker (120 rpm) at 25°C, with irradiances of 30 µmol photons m^−2^ s^−1^on a 12∶12 light dark cycle. Each microalga was grown in triplicate with a total volume of 500 ml. Growth was monitored based on OD_620 nm_; which has a high correlation (r^2^ = 0.9) with chlorophyll a (Chl-a) [30]. In the present study, OD_620 nm_ was strongly correlated to Chl-a content (r^2^ ranged from 0.9102 to 0.9877) for the 16 strains (2 Cyanophytes, 2 diatoms, 14 Chlorophytes) used.

**Table 1:** List of strains used.

Strain	*Habitat	Origin	#Medium
Cyanophyta (Blue-green algae)			
*Synechococcus elongatus* Nageli UMACC 105	FW	Contaminant of *Spirulina* sp. culture	Kos
*Spirulina platensis* (*Arthrospira*)(Gomont)Geitler UMACC 159	FW	Israel	Kos
Chlorophyta (Green algae)			
*Chlorella vulgaris* Beijerinck UMACC 001	FW	Fish pond at IPSP Farm, University of Malaya	BBM
*Scenedesmus* sp. Meyen UMACC 036	FW	Fish tank (Tilapia), IPSP Farm, University of Malaya	BBM
*Scenedesmus quadricauda* (Turpin) Berbisson UMACC 041	FW	Fish tank (Tilapia), IPSP Farm, University of Malaya	BBM
*Chlorella vulgaris* Beijerinck UMACC 051	FW	POME Aerobic pond, Batang Berjuntai, Selangor, Malaysia	BBM
*Scenedesmus* sp. Meyen UMACC 068	FW	Fish tank with goat dung, IPSP Farm, University of Malaya	BBM
*Oocystis* sp. Nageli et A.Braun UMACC 074	FW	Painted Masonry, Science Faculty, University of Malaya	BBM
*Chlorococcum oviforme* Archibald et Bold UMACC 110	FW	Pond at the IPSP farm, University of Malaya	BBM
*Chlorococcum* sp. Meneghini UMACC 207	FW	Plastic container, shop houses, Johor Bahru, Malaysia	BBM
*Chlorella* sp. Beijerinck UMACC 255	M	Sea Bass Pond at Sepang, Selangor, Malaysia	Prov
*Chlorella* sp. Beijerinck UMACC 256	M	Sea Bass Pond at Sepang, Selangor, Malaysia	Prov
*Chlorella* sp. Beijerinck UMACC 258	M	Sea Bass Pond at Sepang, Selangor, Malaysia	Prov
*Chlorella* sp. Beijerinck UMACC 313	FW	POME Anaerobic Pond, Labu Palm Oil Mill, Malaysia	BBM
Bacillariophyta (Diatoms)			
*Coscinodiscus granii* Gough CCMP 1817	M	University of Rhode Island, Rhode Island USA	f/2
*Coscinodiscus wailesii* Gran et Angst CCMP 2513	M	Atlantic Ocean (off Georgia Coast, USA)	f/2

*FW: Fresh water; M: Marine.

# Kos: Kosaric Medium (modified after Zarrouk,1966); BBM: Bold's Basal Medium (Nichols and Bold, 1965);

Prov: Prov Medium (CCMP, 1996); f/2: f/2 Medium (Guillard and Ryther, 1962).

### Algal libraries

#### Growth and biochemical profiling

The specific growth rate (μ, day^−1^) for all cultures were based OD_620 nm_ and calculated using the following formula:
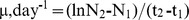
where N_2_ is OD_620 nm_ at t_2;_ N_1_ is OD_620 nm_ at t_1_, and t_2_ and t_1_ are time periods within the exponential phase [31].

The algal samples were harvested at the end of the experiment (stationary phase) by Millipore filtration using glass fibre filter paper (Whatman GF/C, 0.45 µm) for determination of dry weight and extraction of biochemicals. For dry weight (DW) determination, a known volume of the culture was filtered onto an oven-dried pre-weighed glass fibre filter, which was then dried in an oven at 100°C for 24 h. The DW was calculated as follows:
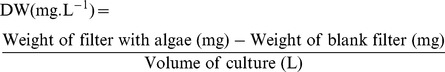



The biomass at stationary phase was determined as dry weight (B_DW_) as well as calculated from the Chl-a content ×67 (B_CHL_), assuming that Chl-a makes up 1.5% of the cell biomass [32], [33]. Protein content of cells was determined by the dye-binding method after extraction in 0.5 N NaOH [34]. Carbohydrates extracted from the cells in 2N HCL were determined using the phenol-sulphuric acid method [35]. Lipids were extracted in MeOH-CHCl_3_-H_2_O (2∶1∶0.8) and determined by gravimetric method [36]. These biochemicals were expressed as %B_CHL_. This may avoid errors with the diatoms that contain silicate frustules but since the content of Chl-a may vary with taxa, these values can only be considered as estimates for comparison between strains.

#### Pulse amplitude modulation (PAM) fluorometer measurement of 16 algal strains

Photosynthetic parameters were measured fluorometrically using a Diving-PAM (Walz, Germany) [37], [21], [38]. Data provided by the PAM based on fluorescence is useful for assessing the performance of a photosynthetic microbial fuel cell. Inglesby et al. [39] showed that in a study using *Arthrospira maxima*, the use of *in situ* fluorescence detection allowed for a direct correlation between photosynthetic activity and current density. Rapid light curves (RLC) were obtained under software control (Wincontrol, Walz). Red light emitting diodes (LEDs) provided the actinic light used in the RLC at the level of 0, 307, 426, 627, 846, 1267, 1829, 2657 and 4264 µmol photons m^−2^ s^−1^. The cultures of each strain were dark-adapted for 15 minutes before exposure to each light level for 10 seconds. Maximum quantum efficiency (F_v_/F_m_), a parameter to indicate the physiological state of phytoplankton was used to indicate if the cells were stressed by the exposure to light: 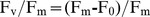 where F_m_ is the maximum fluorescence and F_0_ is the minimum fluorescence resulting in the variable fluorescence F_v_. The maximum photosynthetic efficiency was determined from the initial slope (α) of the RLC. The relative electron transport rate (rETR) was calculated by multiplying the irradiance by quantum yield measured at the end of each light interval. The RLC consists of eight consecutive ten-second intervals of actinic light with increasing intensity. The photoadaptive index (E_k_) is obtained from the curve fitting model [40]. The interception point of the alpha (α) value with the maximum photosynthetic rate (rETRmax) is defined as: 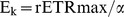. The Non-Photochemical Quenching (NPQ) reflects the ability of a cell to dissipate excess light energy harvested during photosynthesis as heat and is used as an indicator of photoprotection. NPQ is calculated as (F_m_′). 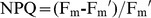. F_m_ is the maximum fluorescence yield during the saturating flash and F_m_′ is the maximum fluorescence in the light-adapted state during the saturating flash. All statistical analyses were performed using the Statistica 8 program. Eight strains were selected according to photosynthetic performance based on F_v_/F_m_, rETRmax, alpha, E_k_ and NPQ. Different algal types and different habitats are also considered as factors that influence the selection of strain.

### Growth and photosynthetic efficiency of algal biofilms

Eight strains namely the Cyanophytes *Synechoccus elongatus* (UMACC 105), *Spirulina platensis* (UMACC 159), the Chlorophytes *Chlorella vulgaris* (UMACC 001), *Chlorella vulgaris* (UMACC 051), *Chlorococcum* sp. (UMACC 207), *Chlorella* sp. (UMACC 256), *Chlorella* sp. (UMACC 313) and the diatom *Coscinodiscus wailesii* (CCMP 2513) were used for the biofilm studies. 100 ml of exponential phase cultures of OD_620 nm_ = 0.5 were used. Each culture was placed into a 200 ml autoclaved glass staining jar. ITO coated glass slides (purchased from KINTEC, Hong Kong) and glass slides measuring 20×20 mm were placed in the staining jar with the microalgae culture and transferred into an incubator at 24°C illuminated by cool white fluorescent lamps (30 µmol m^−2^ s^−1^) on a 12∶12 hour light-dark cycle to allow for the algae biofilms to form on the slides. This experiment was conducted in triplicates. The biofilm growth was monitored by photographing the slide surface with a Sony Cyber-Shot DSC-WX30 Camera every three days until the slides were completely covered by the biofilm. The surface area coverage, SAC (%) of the biofilm captured in the photograph was calculated using ImageJ software [41]. At the end of the experiment (day 15), the biofilm thickness of each slide was measured using Elcometer 3230 Wet Film Wheels [42]. The wheel was held using a finger and thumb by its centre and the wheel was placed on the wet film ensuring that it was perpendicular to the algal film. The wheel was rolled across the algal film through an angle of 180° and then removed from the surface. The thickness of the biofilms was recorded based on the scale on the side of the wheel. The biofilms were removed by washing using jets of distilled water from a pipette, into a sterile beaker for extracting biomass for determination of Chl-a content. The microalgae cells were then harvested by millipore filtration using filter paper (Whatman GF/C, 0.45 µm) and the Chl-a of the eight strains were extracted using acetone [31].The absorption of the extract was measured at 665 nm, 645 nm and 630 nm and the Chl-a content calculated using the formula below:
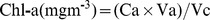
Where,
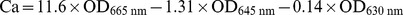


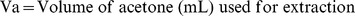


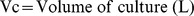


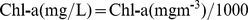



### Pulse amplitude modulation (PAM) fluorometer measurement of biofilms

Photosynthetic parameters were measured fluorometrically using a Diving-PAM (Walz, Germany) as described above [21], [38]. RLC were obtained under software control (Wincontrol, Walz). Red light emitting diodes (LEDs) provided the actinic light used in the RLC at the level of 0, 127, 205, 307, 426, 627, 846, 1267 and 1829 µmol photons m^−2^ s^−1^. The biofilm of each ITO slide on day 15 was dark adapted for 15 minutes prior to the exposure to each light level for 10 seconds.

### BPV set up and electrical measurement

**Figure 1:**
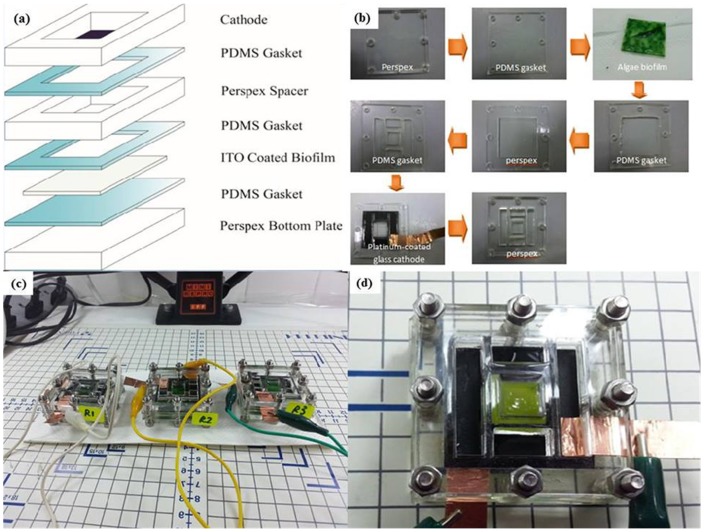
The construction of the Biophotovoltaic (BPV) device (a) Exploded view of a BPV device, (b) Stepwise set-up of the biophotovoltaic device, (c) BPV devices set up in triplicate, (d)Top view of a working BPV device with an algal biofilm formed on an ITO anode.

The BPV devices used in these studies was provided by our collaborators from the University of Cambridge. The closed, single-chamber BPV consisted of a 50×50 mm platinum-coated glass cathode placed in parallel with 35×35 mm ITO coated glass with biofilm grown on the surface (10 mm apart) in a clear Perspex chamber sealed with polydimethylsiloxane (PDMS) and then filled with medium (Figure 1 a–d). The body of the open-air, single-chamber BPV was constructed of clear Perspex [11], [13].

Biofilms of the two strains of Cyanophytes, the *Synechoccus elongatus* (UMACC 105) and *Spirulina platensis* (UMACC 159), and the two Chlorophytes *Chlorella vulgaris* (UMACC 051), and *Chlorella* sp. (UMACC 313) grown on ITO, were placed in the devices and the experiment conducted in triplicates. Crocodile clips and copper wire served as connection between anode and cathode to the external circuit. Prior to operation, the chambers were filled with fresh medium (Bold's Basal medium and Kosaric medium) and maintained at 25°C with irradiance of 30 µmol photons m^−2^ s^−1^ for the duration of the experiments. Current outputs were measured using a multimeter (Agilent U1251B). Polarization curves were generated for each strains by applying different resistance (10 MΩ, 5.6 MΩ, 2 MΩ, 560 KΩ, 240 KΩ, 62 KΩ, 22 KΩ, 9.1 KΩ, 3.3 KΩ and 1.1 KΩ) loads to the external circuit. All experiments were conducted in triplicates.

## Results

### Algae libraries

#### Growth and biochemical profiling

Table 2 shows the growth rates, biochemical characteristics (protein, lipid, carbohydrate) and PAM data of the 16 strains. Specific growth rate ranged from 0.18 to 0.56 d^−1^. The fastest growing algae were the Chlorophytes, *Chlorella* sp. (UMACC 258) (0.58 d^−1^), *Scenedesmus quadricauda* (UMACC 041) (0.37 d^−1^), *Scenedesmus* sp. (UMACC 068) (0.36 d^−1^) and the diatom *Coscinodiscus granii* (CCMP 1817) (0.36 d^−1^). Stationary phase was reached between 9 to 20 days with biomass (B_CHL_) ranging from 139.70±15.70 µg.ml^−1^ (*Chlorella vulgaris* UMACC 051) to 1158.77±30.28 µg.ml^−1^ (*Spirulina platensis* UMACC 159). The lipid, carbohydrate and protein contents were calculated based on biomass (stationary phase culture) from Chl-a (B_CHL_). Lipid content ranged from 24.92±6.07 (*Chlorococcum* sp.) to 63.64±2.20% DW (*Chlorella* sp. UMACC 256). Carbohydrate content ranged from 2.36±0.10% (*Spirulin*a *platensis* UMACC 159) to 18.77±1.58% DW (*Chlorococcum oviforme* UMACC 207). Protein ranged from 14.69±1.18 (*Chlorella* sp. UMACC 256) to 53.74±4.28% DW (*Chlorella vulgaris* UMACC 051). In general, the Chlorophytes, especially strains of *Chlorella*, had higher lipid and protein contents than the diatoms and Cyanophytes.

**Table 2:** Statistical comparison of biochemical profile and PAM data of 16 algal strains, data as means ±S.D. (n = 3).

Strain	Specific Growth Rate (μ,day −1)	*Stat. Phase (day)	Biomass at stationary phase based on dry weight (BDW) (µg/ml)	Biomass at stationary phase based on Chl- a content ×67 (B_CHL_) (µg/ml)	#Lipid (% dry weight)	# δCarbohy. (% dry weight)	#Protein (% dry weight)	rETRmax (µmol photons m^−2^ s^−1^)	Alpha (α)	E_k_ (µmol photons m^−2^ s^−1^)	F_v_/F_m_
*Synechococcus elongatus* UMACC 105	0.25±0.02^b^	17	325.82±23.04^d,e^	208.37±4.39^a^	26.48±1.68^f^	7.44±0.39^a,b^	44.93±3.40^a^	147.50±18.42^a,b,c^	0.31±0.01^e^	473.86±79.05^a^	0.46±0.02^b^
*Spirulina platensis* UMACC 159	0.24±0.02^b^	20	1158.77±30.28^a^	330.76±2.54^a^	53.27±7.45^a,b,c^	2.36±0.10^b^	40.44±9.34^a^	147.61±6.80^a,b,c^	0.39±0.01^e^	380.77±22.74^a,d^	0.43±0.02^b^
*Chlorella vulgaris* UMACC 001	0.18±0.02^d^	10	226.69±13.38^f^	127.52±5.37^f^	45.52±6.21^c,d,e^	4.30±1.59^a,b^	36.87±1.46^a,b,c^	68.53±0.46^e,f^	0.74±0.02^a,b,c,d^	93.20±2.33^f^	0.76±0.02^a^
*Scenedesmus* sp. UMACC 036	0.27±0.02^b^	9	349.92±27.30^c,d^	191.40±4.75^c^	43.28±3.88^c,d,e^	6.32±1.70^a,b^	38.98±5.43^b,c,d^	64.68±11.44^f^	0.84±0.01^a^	84.48±1.46^f^	0.78±0.01^a^
*Scenedesmus quadricauda* UMACC 041	0.37±0.03^c^	10	270.17±16.68^e,f^	123.50±7.85^f^	42.45±6.20^c,d,e^	4.26±0.58^a,b^	35.14±5.62^a,b,c^	80.44±20.78^d,e,f^	0.70±0.10^b,c,d,e^	127.89±54.32^d,e.f^	0.75±0.03^a,b^
*Chlorella vulgaris* UMACC 051	0.25±0.02^c^	14	139.70±15.07^g^	127.52±4.94^f^	34.09±2.47^e,f^	4.31±0.59^a,b^	53.74±4.28^a^	118.21±20.51^b,c,d^	0.49±0.01^c,d,e^	240.26±48.22^c,d,e^	0.60±0.00^a,b^
*Scenedesmus* sp. UMACC 068	0.36±0.03^b^	9	393.67±15.57^b,c^	238.74±3.44^b^	49.09±1.38^b,c,d^	5.92±1.82^a,b^	30.45±6.60^a,b^	71.16±1.98^e,f^	0.76±0.01^a,b,c^	94.08±3.84^f^	0.77±0.01^a^
*Oocystis* sp. UMACC 074	0.34±0.02^c^	9	439.61±18.66^b^	190.95±5.84^c^	41.84±5.11^c,d,e^	7.01±1.73^a,b^	36.87±2.96^a,b,c^	97.91±17.23^d,e,f^	0.85±0.04^a^	115.81±27.06^d,e,f^	0.80±0.02^a^
*Chlorococcum oviforme* UMACC 110	0.34±0.02^b^	10	257.78±19.24^f^	136.90±7.23^e,f^	43.11±4.22^c,d,e^	6.76±1.64^a,b^	38.72±1.88^a,b^	84.82±7.58^d,e,f^	0.82±0.04^a^	104.47±13.80^f^	0.74±0.00^a^
*Chlorococcum* sp. UMACC 207	0.23±0.02^c^	15	240.49±14.18^f^	148.96±4.85^e^	24.92±6.07^f^	18.77±1.58^a^	48.92±2.94^a^	154.07±22.00^a,b,c^	0.51±0.05^b,c,d,e^	302.60±48.08^b,c^	0.57±0.02^a,b^
*Chlorella* sp. UMACC 255	0.33±0.02^b^	9	362.31±23.59^c,d^	119.26±8.23^f^	51.65±1.05^a,b,c^	5.56±0.53^a,b^	26.56±2.68^b,c^	86.60±11.88^d,e,f^	0.78±0.03^a,b,c^	111.69±20.62^d,e,f^	0.76±0.01^a^
*Chlorella* sp. UMACC 256	0.23±0.02^a^	9	352.00±37.28^c,d^	149.86±5.62^e^	63.64±2.20^a^	6.62±0.88^a,b^	15.12±3.28^b,c^	94.06±10.87^d,e,f^	0.88±0.12^a^	108.58±22.07^e,f^	0.77±0.04^a^
*Chlorella* sp. UMACC 258	0.56±0.02^c^	9	337.88±27.85^c,d^	173.08±9.24^d^	58.98±5.04^a,b,c^	3.92±0.20^b^	14.69±1.18^c^	85.51±13.87^d,e,f^	0.80±0.04^a,b^	107.52±23.23^e,f^	0.76±0.01^a^
*Chlorella* sp. UMACC 313	0.33±0.02^b^	14	349.29±15.07^c,d^	194.52±2.05^c^	50.65±4.06^a,b,c^	2.68±1.15^b^	33.17±2.24^a,b,c^	109.98±9.23^c,d,e^	0.45±0.03^d,e^	245.55±31.79^c,d^	0.47±0.04^b^
*Coscinodiscus granii* CCMP 1817	0.36±0.01^b^	14	338.71±18.42^c,d^	78.84±7.35^g^	36.76±0.91^d,e,f^	16.79±2.82^a^	40.87±1.23^a^	161.60±23.41^a,b^	0.41±0.04^e^	403.25±93.92^a,b^	0.45±0.03^b^
*Coscinodiscus wailesii* CCMP 2513	0.33±0.01^c^	15	372.10±12.83^c,d^	94.02±3.93^g^	46.07±2.97^b,c,d,e^	17.35±2.87^a^	31.22±3.24^a,b,c^	166.32±13.57^a^	0.40±0.03^e^	425.18±71.67^a,d^	0.45±0.02^b^

Differences between alphabets indicate significant difference between different strains. (ANOVA, Turkey HSD test, p<0.05). Alpha is photosynthetic efficiency and indicates the amount of ETR per photon. E_k_ is the photoadaptive index and indicates how well cells are adapted to their light environment.

*Stat.: Stationary;

δ Carbohy.;

# Values based on biomass (B_CHL_) calculated from (Chl-a ×67).

#### Pulse amplitude modulation (PAM) fluorometer measurement of 16 strains

Maximum relative electron transport rate (rETRmax) ranged from 64.68 µmol electrons m^−2^ s^−1^ to 166.32 µmol electrons m^−2^ s^−1^. The highest rETRmax were observed from the diatoms *Coscinodiscus wailesii* (CCMP 2513) (166.32 µmol electrons m^−2^ s^−1^) and *Coscinodiscus granii* (CCMP 1817) (161.60 µmol electrons m^−2^ s^−1^), followed by the Chlorophyte *Chlorococcum* sp. (UMACC 207) (154.07 µmol electrons m^−2^ s^−1^) and the two Cyanophytes *Synechococcus elongatus* (UMACC 105) (147.50 µmol electrons m^−2^ s^−1^) and *Spirulina platensis* (UMACC 159). High rETRmax was correlated to high photoadaptive index (E_k_) for all five strains above, but not to the index of light efficiency, α, and the maximum quantum efficiency of PSII (F_v_/F_m_). The strains that reached stationary phase earlier (Chlorophytes) had higher F_v_/F_m_ values than the Cyanophytes and diatoms that took 14 to 20 days to reach stationary phase. The α values varied between 0.31±0.01 (*Synechococcus elongatus* UMACC 105) to 0.88±0.12 (*Chlorella* sp. UMACC 256). The photoadaptive index, E_k_ varied from 84.48±1.46 µmol photons m^−2^ s^−1^ (*Scenedesmus* sp. UMACC 036) to 473.86±79.05 µmol photons m^−2^ s^−1^ (*Synechococcus elongatus* UMACC 105). Maximum quantum yields, F_v_/F_m_ varied from 0.43±0.02 (*Spirulina platensis* UMACC 159) to 0.80±0.02 (*Oocystis* sp. UMACC 074).

**Figure 2:**
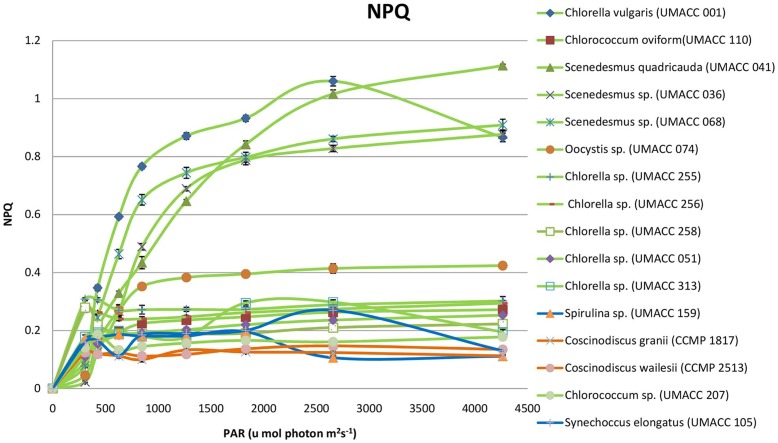
Non-photochemical quenching of sixteen microalgae strains (n = 3).

For all 16 strains, NPQ presented at low irradiances (307 µmol photons m^−2^ s^−1^) (Figure 2). The NPQ values that indicate photoprotection were very low or not observed in both the Cyanophytes and diatoms. Most strains had NPQ values lower than 0.43 except for four Chlorophyte strains. *Scenedesmus* sp. (UMACC 036), *Scenedesmus* sp. (UMACC 068), *Chlorella vulgaris* (UMACC 001) and *Scenedesmus quadricauda* (UMACC 041) that registered highest NPQ values at 0.877, 0.909, 1.06 and 1.114 respectively. *Chlorella vulgaris* (UMACC 001) reached highest NPQ at 2657 µmol photons m^−2^ s^−1^ and showed significant reduction (0.866) at 4264 µmol photons m^−2^ s^−1^, indicating that the photoprotective level started to decrease when coping with extremely high actinic light.

### Growth and photosynthetic efficiency of biofilms

The biofilms were grown on glass and ITO slides in triplicates. Table 3 shows the surface area coverage (% SAC) of biofilms produced by the eight strains on two different substrates (glass and ITO) on day 3, 6, 9, 12 and 15. The % SAC was monitored every three days. Visible growth of the biofilms was already observed on day 3 except for *Coscinodiscus wailesii* (CCMP 2513) which did not show any biofilm growth on both the glass and ITO slides until day 12 and registered the lowest % SAC among the eight strains. After three days of incubation, the Cyanophytes *Synechococcus elongatus* (UMACC 105) and *Spirulina platensis* (UMACC 159) had formed appreciable biofilms on both glass and ITO. Of the Chlorophytes, the *Chlorella* (UMACC 313) from POME pond and the freshwater *Chlorella* (UMACC 051) had high % SAC as well. All strains reached their maximum % SAC after 15 days of inoculation. *Chlorella* sp. (UMACC 313) showed fastest biofilm coverage, having achieved around 80% SAC on day 6 with both glass and ITO. It achieved highest biofilm coverage with 98.18% SAC on glass and 98.69% SAC on ITO on day 15.

**Table 3:** Statistical comparison of surface are coverage (% of eight strains on ITO and glass slides on day 3, 6, 9, 12 and 15, data as means ±S.D. (n = 3).

Strain	Substrate Material	Surface Area Coverage (%)				
		Day 3	Day 6	Day 9	Day 12	Day 15
*Synechococcus elongatus*	Glass	58.74±7.08^g,h,i^	59.70±1.18^g,h,i^	76.25±0.90^e,f,g^	85.90±6.25^b,c,d^	84.68±4.16^b^
UMACC 105	ITO	65.95±6.56^g,h^	77.38±2.82^d,e,f^	81.9±2.12^d,e,f^	82.66±2.78^d,e,f^	96.07±1.69^a^
*Spirulina platensis*	Glass	23.59±3.13^p,q^	34.26±2.39^m,n^	53.97±7.74^i,j,k^	51.51±3.93^i,j,k^	75.56±6.45^c^
UMACC 159	ITO	38.90±1.47^l,m,n^	78.83±1.38^d,e,f^	84.95±2.00^c,d,e^	88.40±1.21^b,c,d^	97.57±0.77^a^
*Chlorella vulgaris*	Glass	14.74±0.79^p,q,r^	29.36±1.77^o,p^	40.79±0.97^l,m,n^	48.77±1.41^i,j,k^	55.83±1.597^d,e^
UMACC 001	ITO	13.58±0.91^q,r^	32.90±1.83^m,n,o^	43.56±1.31^k,l,m^	51.68±0.58^j,k,l^	56.57±2.11^d^
*Chlorella vulgaris*	Glass	44.90±2.12^j,k,l^	63.38±6.19^g,h^	70.65±3.06^f,g^	77.69±1.69^d,e,f^	95.37±1.36^a^
UMACC 051	ITO	44.58±1.19^k,l,m^	62.35±1.83^g,h,i^	70.57±1.45^f,g^	79.21±1.93^d,e,f^	93.32±1.33^a^
*Chlorococcum* sp.	Glass	9.56±0.76q,r	18.42±1.48^p,q,r^	24.61±2.49^o,p^	36.30±1.31^l,m,n^	45.98±1.50^f^
UMACC 207	ITO	9.06±0.32^r,s^	18.19±1.16^p,q,r^	22.41±1.34^o,p,q^	34.05±1.72^m,n^	46.71±1.12^f^
*Chlorella* sp.	Glass	30.78±1.07^o,p^	46.17±10.80^j,k,l^	41.22±1.00^l,m^	46.42±0.58^j,k,l^	49±1.56^e,f^
UMACC 256	ITO	31.59±2.51^m,n,o^	39.36±0.55^l,m,n^	40.66±0.78^l,m,n^	46.06±0.67^j,k,l^	62.79±2.18^d^
*Chlorella* sp.	Glass	66.08±1.51^g,h^	80.28±0.38^d,e,f^	88.19±1.08^b,c,d^	93.71±1.16^a,b,c^	98.18±0.52^a^
UMACC 313	ITO	67.24±0.78^g,h^	80.25±1.41^d,e,f^	89.69±1.00^a,b,c^	94.88±0.75^a,b^	98.69±0.09^a^
*Coscinodiscus wailesii*	Glass	0^s^	0^s^	0^s^	4.83±0.38^r,s^	10.84±1.08^g^
CCMP 2513	ITO	0^s^	0^s^	0^s^	5.37±0.90^r,s^	13.22±1.30^g^

Differences between alphabets indicate significant difference between different strains. (ANOVA, Turkey HSD test, p<0.05).

Table 4 gives details of the biofilms and the PAM data on day 15 of the study. The fluorescence characteristics of the biofilms from the eight strains exposed to a similar range of irradiance were different. The photosynthetic performance was strain dependent. Biomass (B_DW_) ranged from 11.07±2.89 µg/ml (*Coscinodiscus wailesii* CCMP 2513 on ITO) to 180.00±10.00 µg/ml (*Chlorella* sp. UMACC 313 on ITO). The Chl-a content of the biofilms ranged from 0.037±0.006 µg/ml (*Coscinodiscus wailesii* CCMP 2513 on ITO) to 2.533±0.311 µg/ml (*Chlorella* sp. UMACC 313 on ITO). Biofilm thickness ranged from 7±1 µm (*Coscinodiscus wailesii* CCMP 2513 on glass) to 96±2 µm (*Spirulina platensis* UMACC 159 on ITO). The rETRmax varied from 370.334±2.004 to 508.399±17.077 µmol electrons m^−2^ s^−1^. The α values ranged from 0.485±0.001 (*Chlorella* sp. UMACC 313 on glass) to 0.750±0.050 (*Chlorella vulgaris* UMACC 001 on ITO). E_k_ values were all relatively high and ranged from 582.724±12.559 µmol photons m^−2^ s^−1^ (*Chlorella vulgaris* UMACC 001 on ITO) to 761.731±0.716 µmol photons m^−2^ s^−1^ (*Chlorella* sp. UMACC 313 on glass). F_v_/F_m_ ranged from 0.799±0.011 (*Synechococcus elongatus* UMACC 105 on ITO) to 0.857±0.006 (*Chlorococcum* sp. UMACC 207 on glass).

**Table 4:** Statistical comparison of biomass and PAM data of eight strains, on day 15; data as means ±S.D. (n = 3).

Strain	Substrate	Biomass (µg/ml)	Chl-a (µg/ml)	Biofilm Thickness(µm)	rETRmax (µmol electrons m^−2^s^−1^)	Alpha (α)	E_k_ (µmol photons m^−2^s^−1^)	F_v_/F_m_
*Synechococcus elongatus*	Glass	55.00±10.00^d,e^	0.191±0.009^e^	62±8^c^	427.243±14.846^d^	0.569±0.0210^d^	750.481±2.329^a,b,c^	0.810±0.012^b,c,d^
UMACC 105	ITO	86.67±7.64^c^	0.490±0.054^d^	80±5^b^	417.775±5.148^d,e^	0.553±0.009^d,e,f^	755.498±3.135^a,b^	0.799±0.011^d^
*Spirulina platensis*	Glass	70.00±10.00^c,d^	0.301±0.062^d,e^	87±3^b^	466.672±4.530^a,b,c,d^	0.623±0.006^b,c,d^	749.0923±6.839^a,b,c,d^	0.845±0.016^a,b,c^
UMACC 159	ITO	130.00±10.00^b^	0.845±0.028^c^	96±2^a^	452.767±16.133^b,c,d^	0.605±0.024^c,d^	748.029±2.763^b,c,d^	0.828±0.019^a,b,c,d^
*Chlorella vulgaris*	Glass	23.33±2.89^g,h^	0.048±0.003^e^	17±1^e,f^	430.730±32.745^d^	0.732±0.054^a^	588.41±6.445^f^	0.826±0.002^a,b,c,d^
UMACC 001	ITO	35.00±5.00^e,f,g^	0.058±0.006^e^	20±2^e,f^	437.444±38.471^d^	0.750±0.050^a^	582.724±12.559^f^	0.831±0.012^a,b,c,d^
*Chlorella vulgaris*	Glass	50.00±5.00^d,e^	0.302±0.205^d,e^	24±1^e^	420.458±32.686^d,e^	0.562±0.047^d,e^	748.377±4.590^b,c,d^	0.824±0.019^a,b,c,d^
UMACC 051	ITO	151.67±10.41^b^	0.501±0.044^d^	38±3^d^	429.798±1.716^d^	0.573±0.004^d^	750.528±1.612^a,b,c^	0.851±0.004^a,b^
*Chlorococcum* sp.	Glass	26.67±2.89^f,g,h^	0.075±0.011^e^	12±1^f,g,h^	457.495±10.938^a,b,c,d^	0.620±0.015^b,c,d^	737.498±1.133^c,d,e^	0.857±0.006^a^
UMACC 207	ITO	26.67±7.64^f,g,h^	0.087±0.007^e^	15±1^e,f,g,h^	446.736±15.241^c,d^	0.606±0.021^c,d^	736.798±0.770^d,e^	0.840±0.013^a,b,c,d^
*Chlorella* sp.	Glass	46.67±5.77^e,f^	0.184±0.008^e^	15±1^f,g,h^	498.801±14.443^a,b,c^	0.684±0.021^a,b^	729.266±1.226^e^	0.802±0.021^c,d^
UMACC 256	ITO	53.33±5.77^d,e^	0.192±0.006^e^	16±1^e,f,g^	508.399±17.077^a^	0.694±0.021^a,b^	732.519±2.837^e^	0.825±0.018^a,b,c,d^
*Chlorella* sp.	Glass	146.67±7.64^b^	1.364±0.056^b^	82±3^b^	369.186±0.407^e^	0.485±0.001^f^	761.731±0.716^a^	0.819±0.006^a,b,c,d^
UMACC 313	ITO	180.00±10.00^a^	2.533±0.311^a^	87±3^b^	370.334±2.004^e^	0.487±0.004^e,f^	760.447±1.825^a,b^	0.827±0.006^a,b,c,d^
*Coscinodiscus wailesii*	Glass	13.33±2.89^g,h^	0.037±0.006^e^	7±1^h^	501.960±0.480^a,b^	0.681±0.001^a,b,c^	737.454±0.805^c,d,e^	0.854±0.030^a,b^
CCMP 2513	ITO	11.67±2.89^h^	0.037±0.006^e^	8±0^g,h^	502.239±0.421^a,b^	0.681±0.001^a,b,c^	737.503±0.828^c,d,e^	0.834±0.004^a,b,c,d^

Differences between alphabets indicate significant difference between different strains. (ANOVA, Turkey HSD test, p<0.05). Alpha is photosynthetic efficiency and indicates the amount of ETR per photon. E_k_ is the photoadaptive index and indicates how well cells are adapted to their light environment.

**Figure 3:**
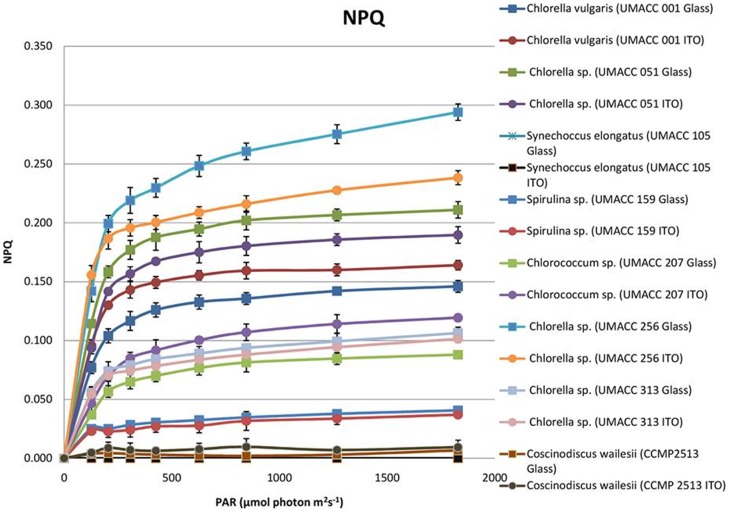
Non-photochemical quenching of biofilms formed by eight strains grown on ITO and glass slides on day 15 (n = 3).

NPQ was active in most of the strains except for the Cyanophyte *Synechococcus elongatus* (UMACC 105) which failed to produce any NPQ during the RLC (Figure 3). NPQ increased markedly and was almost linear following the increase of irradiance. Most of the strains started to produce NPQ at 127 µmol photons m^−2^ s^−1^, with values ranging from 0.004 to 0.156. Strains such as *Chlorella* sp. (UMACC 256), *Chlorella vulgaris* (UMACC 051), *Chlorella vulgaris* (UMACC 001) and *Chlorococcum* sp. (UMACC 207) developed moderate to high levels of NPQ. *Chlorella* sp. (UMACC 256) biofilm grown on glass produced the highest NPQ value of 0.294 while the diatom *Coscinodiscus wailesii* (CCMP 2513) developed the lowest NPQ (<0.01).

### BPV set up and electrical measurements

**Figure 4:**
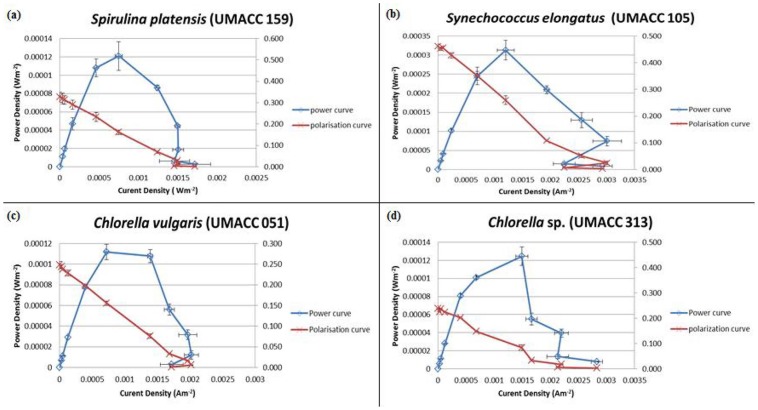
Polarization curves observed for (a) Spirulina platensis (UMACC 159), (b) Synechococcus elongatus (UMACC 105), (c) Chlorella vulgaris (UMACC 051), and (d) Chlorella sp.(UMACC 313).

A study was carried out to correlate the %SAC of biofilm on ITO with the amount of photocurrent being generated as well as the overall performance of the device. Figure 4 shows a polarization curve observed for *Chlorella vulgaris* (UMACC 051), *Chlorella* sp. (UMACC 313), and the Cyanophytes *Spirulina platensis* (UMACC 159) and *Synechococcus elongatus* (UMACC 105). Significant power outputs were seen from all strains. The Cyanophytes *Synechococcus elongatus* UMACC105 (461 mV) and *Spirulina platensis* UMACC 159 (327 mV) produced highest cell voltage at open circuit, followed by the Chlorophytes *Chlorella vulgaris* UMACC 051 (249 mV) and *Chlorella* sp. UMACC 313 (239 mV). Meanwhile, the value of maximum power density in descending order was *Synechococcus elongatus* UMACC 105 (3.13×10^−4^ Wm^−2^)>*Chlorella* sp. UMACC 313 (1.24×10^−4^ Wm^−2^)>*Spirulina platensis* UMACC 159 (1.21×10^−4^ Wm^−2^)>*Chlorella vulgaris* UMACC 051 (1.12×10^−4^ Wm^−2^). As for the value of maximum current density, a descending order, *Synechococcus elongatus* UMACC 105 (2.93×10^−3^ Am^−2^)>*Chlorella* sp. UMACC 313 (2.83×10^−3^ Am^−2^)>*Chlorella vulgaris* UMACC 051 (2.02×10^−3^ Am^−2^)>*Spirulina platensis* UMACC 159 (1.72×10^−3^ Am^−2^) was observed.

## Discussion

The preliminary screening of 16 strains, comprising 14 local strains belonging to the blue-green and green algae, and two diatoms from the CCMP, allowed the selection of eight out of the 16 strains that were characterized in terms of growth rate, biochemical composition (protein, lipid and carbohydrate contents) and photosynthetic efficiency, for further studies on biofilm formation. The 16 strains were cultured till stationary phase to observe the batch culture characteristics. At stationary phase, the lipid contents are expected to be higher than at exponential phase of culture, as lipids are accumulated upon reaching stationary phase, as opposed to protein [30]. This was clearly observed with most of the strains except for the Cyanophyta (*Synechoccus elongatus* UMACC 105) and two Chlorophytes (*Chlorella vulgaris* UMACC 051 and *Chlorococcum* UMACC 207), where protein was highest at day 15. In general, the Chlorophytes contained higher lipid than the other strains, with *Chlorella* sp. (UMACC 256) having the highest lipid (63.64%DW) contents on day 15.

Eight out of the 16 strains, namely two Cyanophytes, five Chlorophytes and one diatom, were selected for the biofilm studies. The Cyanophytes were selected because blue-green algae produce abundant mucilage and are expected to form biofilms easily. The blue-green algae has higher potential to form a hydrated polymeric matrix termed extracellular polymeric substances (EPS) which encourages the biofilm formation [15]. Also from the preliminary screening, the *Synechococcus elongatus* (UMACC 105) and *Spirulina platensis* (UMACC 159) had high rETRmax values, indicating efficient photosynthesis. *Chlorella vulgaris* (UMACC 001) has been used for many studies in our laboratory and has shown ability to grow in wastewaters [43], [44], [45] and has potential for biofuel production [30]. In 2007, Wong and co-workers [46] compared the tolerance of Antarctic, tropical and temperate microalgae to ultraviolet radiation (UVR) stress. When the *Chlorella vulgaris* (UMACC 001) was exposed to ambient light, the specific growth rate was 0.16 d^−1^ but decreased to 0.04 d^−1^ when exposed to ultraviolet radiation (UVR) treatment. Vejesri et al. [30] reported that *Chlorella vulgaris* (UMACC 001) was a potential feedstock for biodiesel production due to high specific growth rate (μ = 0.42 d^−1^) and high saturated fatty acid (SFA) content (68.2%DW). Chu and co-workers [43] reported that immobilized cultures of *Chlorella vulgaris* (UMACC 001) in alginate removed 48.9% of color from textile wastewater. *Chlorella vulgaris* (UMACC 001) showed high NPQ and strong photoprotection with higher capacity to cope with high irradiance. *Chlorococcum* (UMACC 207) had high rETRm as well as high protein (30.29%DW) content. *Chlorella* sp. (UMACC 313) and *Chlorella vulgaris* (UMACC 051) isolated from the aerobic pond for palm oil mill effluent (POME) treatment were selected because they formed biofilms on the base and sides of the culture flasks, and had very high lipid content. One marine alga *Chorella* sp. (UMACC 256) was included because it had the highest rETRmax value among the three marine strains. It was also shown that this strain had high specific growth rate (0.75 d^−1^) and high saturated fatty acid (SFA) content (53.8%DW) in our previous study [30]. One strain from the CCMP culture collection was also selected for comparison with the UMACC cultures. The strain, *Coscinodiscus wailesii* (CCMP 2513) was a centric diatom with high rETRmax (166.32 µmol electrons m^−2^ s^−1^) and higher lipid content of the two diatoms. In addition to the photosynthetic parameter of rETRmax, consideration was also given to the biofuel potential of the strains, in terms of lipid content.

In the biofilm studies, strains like *Synechococcus elongatus* (UMACC105) and *Chlorella* (UMACC 313) were observed to have formed appreciable biofilms after only three days. This may be based on the high production of EPS by *Synechococcus elongatus* (UMACC 105) and the high growth rate of *Chlorella* (UMACC 313). Application of PAM fluorometry in the study of algae biofilms has provided valuable information on the operation of algal BPV platforms. One of the most important information is the non-destructive generation of light-response curves of photosynthetic activity [47]. The distance between the optical fiber-optics and the sample surface was set at 2 mm [48]. Eight selected strains starting with the same concentration of pure cultures at OD_620 nm_ at 0.5. All cultures were transferred into the same incubator at 24°C illuminated by cool white fluorescent lamps (30 µmol m^−2^ s^−1^) on a 12∶12 hour light-dark cycle to ensure that the only variable was the strain of algae in the biofilm development. E_k_ values were all relatively high and ranged from 582.724 to 761.731 µmol photons m^−2^ s^−1^ indicating that all biofilms were able to adapt to the high light intensities (307–1829 µmol photons m^−2^ s^−1^). Under high irradiances, an inverse relationship between NPQ and rETRmax was observed in all strains; higher NPQ values being matched by lower rETRmax values. The Chl-a content of the eight strains increased with the thickness of biofilms. The *Chlorella* species (UMACC 313 and UMACC 051) isolated from the aerobic pond used in POME treatment formed significant biofilms on day 3 on both substrate materials. However, the % SAC of the biofilm from the diatom *Coscinodiscus wailesii* (CCMP 2513) was low compared to other strains probably due to the need for longer time to form hydrated polymeric matrix. Even in the suspension culture during the preliminary screening, this diatom took 15 days to reach stationary phase (Table 2). The substrate effect was minimal as there was no significant difference of % SAC of all strains on both glass and ITO slides, except for the filamentous blue-green alga *Spirulina platensis* (UMACC 159) which grew better on ITO slides compared to glass slides with higher % SAC, Chl-a content and biofilm thickness. From this study, four strains, the Chlorophytes *Chlorella* (UMACC 051, UMACC 313) and the Cyanophytes *Synechococcus elongatus* (UMACC 105) and *Spirulina platensis* (UMACC 159) were identified to produce the most stable biofilms on ITO. The growth and viability of these microorganisms in a working BPV device would have a significant impact on the photocurrent that would be generated [13].

The biofilm samples were dark adapted for at least 15 minutes before PAM measurement. When cultures were placed in the dark, minimum (F_o_) and maximum (F_m_) fluorescence both increased, as did the maximum PSII quantum efficiency (F_v_/F_m_). After dark adaption, the reaction centres were in a relaxed stage, and ready to receive light for photosynthesis [19]. The maximum quantum yield F_v_/F_m_ was obtained when all reaction centres were opened and was proportional to the fraction of reaction centres capable of converting absorbed light to photochemical energy [49]. F_v_/F_m_ is often used as an indicator of photosynthetic capacity. Various studies have reported values of F_v_/F_m_ ranging between 0.1 to 0.65 for natural populations of microalgae [50]. Results in the present study indicated that most samples were in a healthy condition, as the F_v_/F_m_ values were all above 0.800 except for *Synechococcus elongatus* UMACC 105 with a F_v_/F_m_ value of 0.799. Serôdio et al. [51] reported rapid light-response curves of chlorophyll fluorescence in benthic microalgae from a mesotidal estuary located in the west coast of Portugal. The values of photosynthetic parameters were as follows: alpha (α): 0.249–0.473, rETRmax (µmol electrons m^−2^ s^−1^): 118.9 to 424.0, E_k_ (µmol photons m^−2^ s^−1^): 474.1 to 821.4. A similar study carried out by Cartaxana et al. [52] on the same area reported PAM fluorometry as a tool to assess photophysiology of intertidal microphytobenthic biofilms. The microphytobenthos communities were dominated by diatoms, resulting in the following photosynthetic parameters; alpha (α): 0.657, rETRmax (µmol electrons m^−2^ s^−1^): 331, E_k_ (µmol photons m^−2^ s^−1^): 582.72–761.73. In our study, the range of photosynthetic parameters for suspension cultures of the 16 strains were: alpha (α): 0.31–0.91, rETRmax (µmol electrons m^−2^ s^−1^): 64.68–166.32, E_k_ (µmol photons m^−2^ s^−1^): 72.73–473.85 and F_v_/F_m_: 0.43–0.80. For biofilms of the eight strains, the range of photosynthetic parameters were: alpha (α): 0.48–0.75, rETRmax (µmol electrons m^−2^ s^−1^): 369.19–508.40, E_k_ (µmol photons m^−2^ s^−1^): 582.72–760.45 and F_v_/F_m_: 0.80–0.85. Our results showed that algal biofilms have better photosynthetic performance than suspension cultures. Results showed that the algal strains used in this study have high photosynthetic efficiency indicating their potential for use in BPV platforms. In addition, high rETRmax and E_k_ values obtained from the present study reflected an adequate adaptation to high irradiance [53], and was observed in the two Cyanophytes (*Synechococcus elongatus* UMACC 105) and the diatom (*Coscinodiscus wailesii* CCMP 2513). The NPQ values for each strain were compared and an increase in NPQ with changing light intensities was observed in all cases of the biofilms (Figure 3). The change in NPQ is expected due to photoprotection of photosystem II to avoid light induced damage [54]. In the case of the Cyanobacteria (Cyanophyta), the resulting changes in NPQ have been reported to be triggered by the light activation of orange carotenoid protein found in phycobilisomes [55]. In comparison, the NPQ in diatoms is mediated by the light-harvesting complex stress-related (LHCSR) protein and the conversion of diadinoxanthin (Ddx) to diatoxanthin (Dtx) [56]. In green algae (Chlorophyta), the controlled change in pH and its effect on the xanthophyll cycle has a predominated effect on NPQ [54]. In the present study, the Chlorophyte strains were observed to have higher ability (higher NPQ values) to photoprotect themselves than the Cyanophytes and diatoms. The Cyanophytes (blue-green algae) were observed to have the lowest value or in some cases, no NPQ, indicating that they have the lowest capacity for photoprotection. There was no clear correlation between the F_v_/F_m_ values and the NPQ values of the 16 strains in the screening experiment as well as the eight strains used for the biofilm study, except where the lowest F_v_/F_m_ values of the two Cyanophytes also corresponded to the lowest NPQ values (Tables 2 & 4 and Figures 2 & 3). Interestingly the low NPQ values of the two Cyanophytes and two diatoms corresponded to higher E_k_ values, and may indicate that these strains may in fact be tolerant of higher irradiance. This however, has to be confirmed by detailed studies on the relationship between the various parameters. In this study, the four strains, Cyanophytes *Synechococcus elongatus* UMACC 105, *Spirulina platensis* UMACC 159 and Chlorophytes *Chlorella* UMACC 051 and UMACC 313, were selected for the BPV study based on strong ability to form biofilms; however their NPQ ranged from low to high values, to allow the correlation of NPQ to photo current production in a working BPV device. The diatoms had high rETRmax but very low NPQ, and were not selected due to poor ability to form biofilms. On analysis of data from the BPV studies, it was apparent that the Cyanophyte *Synechococcus elongatus* (UMACC 105), was able to give highest performance in the BPV device but it had the lowest NPQ value. The maximum power density was similar for the other Cyanophyte *Spirulina* sp. (UMACC159) and two Chlorophytes *Chlorella* (UMACC 051 and 313), which had higher NPQ. Further studies would need to be carried out to correlate the NPQ of the strains with the ability to extract charge from biofilms in a working BPV device.

All four strains, the Cyanophytes *Synechoccus elongatus* (UMACC 105) and *Spirulina platensis* (UMACC 159), and the Chlorophytes *Chlorella vulgaris* (UMACC 051) and *Chlorella* sp. (UMACC 313), showed ability to produce electrical power outputs (Figure 4 a–d). *Synechococcus elongatus* (UMACC 105) showed higher power outputs compared to other strains. This may be due to the fact that *Synechococcus elongatus* (UMACC 105) registered a high rETRmax values (147.50 µmol electrons m^−2^ s^−1^), high E_k_ value and readily produced biofilms (84.68% SAC on ITO) which were able to accumulate a high level of charge. The *Synechococcus* produces unicells of small dimensions and with production of EPS, would form compact biofilms on the anode, compared to the filamentous *Spirulina* which like the *Arthrospira* used in the study by Inglesby et al. [39], formed porous biofilms. The maximum power density obtained in this study ranged from 1.12×10^−4^ to 3.13×10^−4^ Wm^−2^. Inglesby et al. [39] reported very low power densities from 6.7 to a maximum of 24.8 µW m^−2^ with a similar BPV device using biofilm of the Cyanophyte *Arthrospira maxima* on ITO as well, considering that the theoretical maximum power density achievable when using microalgae in microbial fuel cells is 2.8 Wm^−2^. The low power density obtained in the present study reflects an unoptimised system with the results aimed only for comparison between the four strains. Optimisation of the process in terms of biofilm thickness and irradiance properties may improve the power output. Optimisation of temperature and light intensity in the BPV device may enhance power output, as was shown by Inglesby et al. [39], where an increase of temperature from 25 to 35°C increased power output from 9.9 to 24.8 µW m^−2^ while there may be a limitation to how high the light intensity can be, due to photoinhibition. In the present study. Based on power output per Chl-a content, the four strains can be ranked as follows: *Synechococcus elongatus* (UMACC 105) (6.38×10^−5^ Wm^−2^/µgChl-a)>*Chlorella vulgaris* (UMACC 051) (2.24×10^−5^ Wm^−2^/µgChl-a)>*Chlorella* sp. (UMACC 313) (1.43×10^−5^ Wm^−2^/µgChl-a)>*Spirulina platensis* (UMACC 159) (4.90×10^−6^ Wm^−2^/µgChl-a). These values were obtained by dividing the maximum power density by the total Chl-a extracted from the whole algal biofilm on the ITO slides. However no correlation (P<0.05) was observed between power output and Chl-a content. Of the four strains, *Chlorella vulgaris* (UMACC 051) had highest lipid (31.09% DW) and crude protein (46.70% DW) contents, indicating additional advantages over the other strains. Although it is premature to speculate on the long-term usage of algal cultures in BPV devices, on a large scale, biomass may be generated continuously in the BPV. The biomass may be harvested on a regular basis to prevent over-buildup of the biofilm, which results in reduced bioactivity at the surface of the biofilm furthest from the anode. Photosynthetically efficient strains with high lipid and protein productivities, may have an added advantage over other strains, providing a valuable biomass that may enter an alternative energy-producing system like biodiesel production. This may overcome the lower energy output from algal BPV systems compared to commercially available photovoltaics [39]. However the additional cost of continuous nutrient supply to the biofilms on the anodes and cost of removal of surplus biomass from the biofilms may necessitate innovative design and operation of the BPV devices.

## Conclusions

The present study indicated that local algal strains were good candidates for utilization in BPV platforms in future. According to our screening results, all eight strains were able to form biofilms on ITO anode surfaces with good photosynthetic performance. From this, four strains were selected for the BPV studies, based on their high photosynthetic performance and ability to produce biofilms. The Chlorophytes *Chlorella* species (UMACC 051, UMACC 313) and the Cyanophytes, *Spirulina platensis* (UMACC 159) and *Synechococcus elongatus* (UMACC 105), demonstrated exoelectrogenic activity and showed their capacity to produce significant electrical power outputs without the requirement of additional organic fuel. More work is required to further understand the mechanisms of harnessing light energy and converting them to electricity as well as to investigate the correlation between PAM data and the BPV power output.
